# Forming mechanism of equilibrium and non-equilibrium metallurgical phases in dissimilar aluminum/steel (Al–Fe) joints

**DOI:** 10.1038/s41598-021-03578-0

**Published:** 2021-12-20

**Authors:** Shun-Li Shang, Hui Sun, Bo Pan, Yi Wang, Adam M. Krajewski, Mihaela Banu, Jingjing Li, Zi-Kui Liu

**Affiliations:** 1 Department of Materials Science and Engineering, Pennsylvania State University, University Park, PA 16802 USA; 2 Department of Industrial and Manufacturing Engineering, Pennsylvania State University, University Park, PA 16802 USA; 3 Department of Mechanical Engineering, University of Michigan, Ann Arbor, MI 48109 USA

**Keywords:** Mechanical engineering, Metals and alloys, Electronic structure

## Abstract

Forming metallurgical phases has a critical impact on the performance of dissimilar materials joints. Here, we shed light on the forming mechanism of equilibrium and non-equilibrium intermetallic compounds (IMCs) in dissimilar aluminum/steel joints with respect to processing history (e.g., the pressure and temperature profiles) and chemical composition, where the knowledge of free energy and atomic diffusion in the Al–Fe system was taken from first-principles phonon calculations and data available in the literature. We found that the metastable and ductile (judged by the presently predicted elastic constants) Al_6_Fe is a pressure (*P*) favored IMC observed in processes involving high pressures. The MoSi_2_-type Al_2_Fe is brittle and a strong *P*-favored IMC observed at high pressures. The stable, brittle η-Al_5_Fe_2_ is the most observed IMC (followed by θ-Al_13_Fe_4_) in almost all processes, such as fusion/solid-state welding and additive manufacturing (AM), since η-Al_5_Fe_2_ is temperature-favored, possessing high thermodynamic driving force of formation and the fastest atomic diffusivity among all Al–Fe IMCs. Notably, the ductile AlFe_3_, the less ductile AlFe, and most of the other IMCs can be formed during AM, making AM a superior process to achieve desired IMCs in dissimilar materials. In addition, the unknown configurations of Al_2_Fe and Al_5_Fe_2_ were also examined by machine learning based datamining together with first-principles verifications and structure predictions. All the IMCs that are not *P-*favored can be identified using the conventional equilibrium phase diagram and the Scheil-Gulliver non-equilibrium simulations.

## Introduction

Joining of dissimilar materials has become increasingly important to create lightweight, high-performance, and economic structures employed in various industries, for example, automotive^1^, aerospace^2,3^, marine^4^, and information technology^5^. Specially, joining of aluminum (Al) to steel/iron (Fe) is of eminent technical interest due to the potential use of two essential engineering materials in the same design^1,6^. It is known that mechanical properties of dissimilar materials are strongly affected by the type, amount/thickness, and morphology of metallurgical phases formed at the bonding interfaces. For example, the formation of brittle intermetallic compounds (IMCs), such as η-Al_5_Fe_2_^7,8^, is usually detrimental to the performance of dissimilar materials joints owing to the reduction of materials’ strength, ductility, and fracture toughness. A great deal of effort in chemistry and process design is hence required to avoid or reduce their formation in dissimilar materials, demanding fundamental understanding of phase stability of IMCs during various processes, for example, different pressure (*P*) and temperature (*T*) profiles under a given chemical composition.

**Figure 1:**
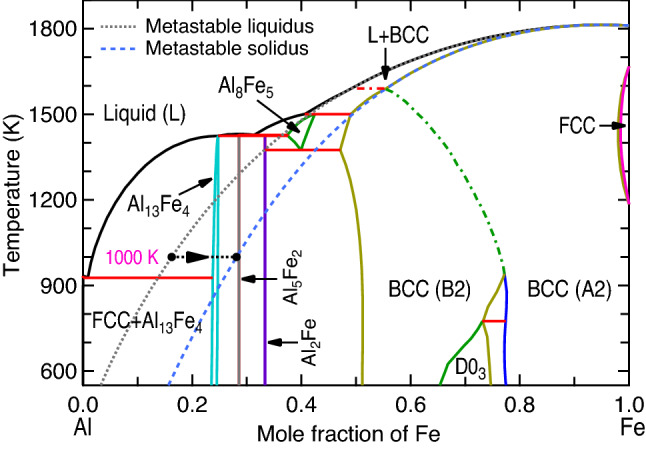
Calculated Al–Fe phase diagram based on CALPHAD modeling by Sundman et al.^9^. The metastable liquidus and solidus lines are plotted to analyze the formation of non-equilibrium phases from the supersaturated solution phase. One example at 1000 K is shown for phase equilibrium from the supercooled liquid with composition *x*_Fe_ = 0.163 to the supersaturated BCC phase with *x*_Fe_ = 0.281, then the composition 0.281 is used to calculate thermodynamic driving forces of IMCs at 1000 K; see Fig. 6.

**Figure 2:**
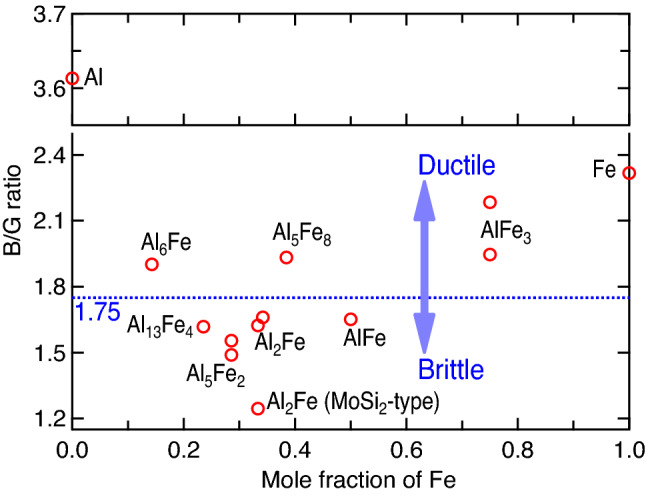
Calculated bulk modulus versus shear modulus (*B*/*G*) ratios of Al–Fe IMCs based on the present DFT calculations; see details in Table S3. Note that Pugh’s criterion^11^ of 1.75 is a rough value to separate the ductile and brittle materials, as discussed in the authors’ responses to Reviewers in Ref. 12.

Relevant to the present focus of Al–Fe joints, there are six IMCs shown in the equilibrium Al–Fe phase diagram under external pressure *P* = 0 GPa; see Fig. 1, which was modelled by the CALPHAD (calculations of phase diagram) approach by Sundman et al.^9^. It includes the stable IMCs of θ-Al_13_Fe_4_, η-Al_5_Fe_2_, Al_2_Fe, AlFe (in B2 structure), AlFe_3_ (D0_3_), and the metastable ε-Al_8_Fe_5_ (D8_2_). In addition, the other metastable IMCs include Al_6_Fe and Al_m_Fe (4 ≤ m ≤ 4.4)^10^, which are absent in Fig. 1. It is believed that the Al-rich IMCs (Al_13_Fe_4_, Al_5_Fe_2_, and Al_2_Fe) are brittle and favor crack nucleation in the joints, while the Fe-rich IMCs (i.e., the BCC based AlFe and AlFe_3_) show higher ductility and strength^7,8^. The ductility and brittleness of these IMCs are shown in Fig. 2 according to Pugh’s criterion^11,12^, i.e., the ratio of bulk modulus versus shear modulus (*B*/*G*) based on the present first-principles calculations (*cf.*, “Details of first-principles calculations” section). It indicates the ductile Al_6_Fe, Al_5_Fe_8_, and AlFe_3_; the less ductile Al_13_Fe_4_ and AlFe; and the brittle Al_5_Fe_2_ and Al_2_Fe. Table 1 summarizes the Al–Fe IMCs formed in different processes reported in the literature. The metastable, ductile Al_6_Fe was observed in the processes of direct chill casting (example #1 in Table 1)^10^, high-pressure die casting (#2)^13^, equal channel angular extrusion (#3)^14^, tungsten inert gas (TIG) welding-brazing (#4)^15^, and additive manufacturing (AM) via laser powder bed fusion (#5)^16^. These observations suggest that Al_6_Fe is an IMC existing at high pressures. Table 1 further depicts that most of the stable and even metastable Al–Fe IMCs were observed in AM processes. For example, Al_6_Fe, Al_13_Fe_4_, Al_2_Fe, Al_5_Fe_2_, AlFe, and/or AlFe_3_ were formed during the processes of laser powder bed fusion^16^, laser cladding^17^, direct energy deposition^18^, laser metal deposition^19^, and/or wire-arc AM^20,21^ (see examples #5 to #10 in Table 1). In particular, the ductile (or less brittle) Al_13_Fe_4_, AlFe, and AlFe_3_^20–22^ (examples #9 to #11) were observed in Al–Fe based functional graded materials fabricated by additive manufacturing. These experiments indicate that AM is an exceptional process to tailor compositions and in turn the desired IMCs. Note that the AM induced residual stress is usually less than 1 GPa, for example, 290–416 MPa in 304L stainless steel^23^, and up to ~ 660 and 200 MPa for tensile and compressive, respectively, in 316L stainless steel^24^. These stresses are usually negligibly small to induce solid state phase transition. In the fusion and/or solid-state welding joints, Al_5_Fe_2_ is the most observed IMC (usually adjacent to iron/steel) followed by Al_13_Fe_4_ (usually adjacent to Al) processed by, for example, laser welding^25–27^ (see examples #13 to #15 in Table 1), friction-type solid state welding^28–36^ (#16 to #24), cold metal transfer fusion welding^37^ (#25), and double electrode gas metal arc welding^38^ (#26). The other IMCs such as Al_2_Fe and AlFe_3_ were also observed in welding processes, depending on welding conditions (e.g., energy inputs)^7^; see examples #14, #21, and #22 in Table 1. The same as those in welding processes, Al_5_Fe_2_ (majorly) and Al_13_Fe_4_ were also observed in immersion testing with solid Fe and liquid Al^39–41^ (see examples #27 to #29 in Table 1), Al–Fe diffusion couples^42–44^ (#30 to #32), high-temperature reactive sintering^45^ (#12), and aluminized steel^46^ (#33).

**Table 1:** Experimentally observed Al–Fe IMCs in various processes in the literature. Note that the compositions quoted in this table are in wt.% unless otherwise stated.

#, Ref	Materials	Methods	Observed Al–Fe IMCs
1^10^	Sheet ingots of Al alloys: 1050, − 1100, and − 5005	Direct-chill casting	Al_13_Fe_4_ with cooling rate < 3 K/s; Al_6_Fe with cooling rate from 1–3 to 10–20 K/s; and Al_m_Fe (4 ≤ m ≤ 4.4) with cooling rate > 20 K/s
2^13^	Al-5Mg-*x*Fe-0.6Mn (*x* = 0.1–2 wt.%)	High pressure die casting at 720 °C	Al_6_(Fe,Mn) and Al_13_(Fe,Mn)_4_^a^
3^14^	Al-3Fe alloy	Equal channel angular extrusion at room temperature	Al_6_Fe
4^15^	Al alloy 5A06 and SUS321 steel sheets	Tungsten inert gas welding-brazing with Al filler	Al_6_Fe in welded seam with Nocolok flux; and Al_13_Fe_4_ in the IMC layer
5^16^	Al-2.5wt.% Fe alloy powders	Laser powder bed fusion (LPBF)	Al_13_Fe_4_ (reduced in the LPBF samples compared to ingots) and Al_6_Fe
6^17^	Al and Fe powders	Layer by layer laser cladding	Al_2_Fe (with 34–52 at. % Al cases); and Al_5_Fe_2_ and Al_13_Fe_4_ (with > 52 at. % Al cases)
7^18^	Al and Fe powders	Direct energy deposition (DED) type process	AlFe_3_ (for composition Fe-28Al); AlFe_3_ + AlFe (for Fe-36Al); and AlFe (for Fe-50Al)
8^19^	Fe-28Al and Al powders	Laser metal deposition for graded Fe-Al/steel samples and heat treated at 700 °C	AlFe and Al_2_Fe (cracks originated in Al-rich part) followed by Al-rich AlFe below
9^20^	Al and Fe wires	Wire-arc AM (WAAM) for Fe-AlFe functionally graded material (FGM)	AlFe_3_ and AlFe
10^21^	Al and Fe wires	WAAM to fabricate Fe-rich IMC (25 at.% Al)	AlFe_3_
11^22^	Pure Al with Al-10 wt.% Fe	Vacuum centrifugal method to make Al-Al_13_Fe_4_ FGM	Al_13_Fe_4_
12^45^	Compressed mixture of Al and Fe powders	High-temperature reactive sintering (800 and 900 °C)	Al_5_Fe_2_ and Al_2_Fe; and AlFe (long-term annealing)
13^25^	Al-steel overlap joints	Laser welding (up to 1200 °C)	Al_5_Fe_2_ (assuming diffusion from Fe to Al only)
14^26^	Al alloy 6061-T6 and galvanized steel DP590	Laser welding without filler	Al_13_Fe_4_ and Al_5_Fe_2_ with linear energy density of 162 J/mm; Al_13_Fe_4_, Al_5_Fe_2_, and AlFe with 309 J/mm
15^27^	Al alloy 5083 and low alloy steel (XF350) plates	Fiber laser welding with 8 kW of max power	Al_5_Fe_2_ near steel (main) and Al_13_Fe_4_ near Al
16^28^	Pure Al (1100) and low carbon steel	Friction stir welding	Al_5_Fe_2_ and Al_13_Fe_4_
17^29^	Al alloy (5186) and low carbon steel	Friction stir welding	Al_5_Fe_2_ (adjacent to Fe) and Al_13_Fe_4_ (adjacent to Al, facilitated by Fe diffusion)
18^30^	Al sheet (6061) and galvannealed steel sheet	Friction stir welding	Al_13_Fe_4_ (large size, diffusion induced) and AlFe_3_ (small size)
19^31^	Al alloy 5754 with coated DP600 or 22MnB5 steel	Diffusion bonding by friction stir welding	Al_5_Fe_2_ in low welding speeds (16 mm/min) and AlFe in 45 mm/min
20^32^	Al alloy 5083 and steel (< 0.1 wt.% C) sheets	Annealing of friction stir lap welds	Al_5_Fe_2_ (major) and Al_13_Fe_4_ annealed at 673 K for 180 min
21^33^	Al alloy 6061-T6 and AISI 1018 steel	Friction welding	Al_5_Fe_2_ and AlFe (suggested based on compositions)
22^34^	Al sheet (6016) and galvanized IF-steel sheet	Friction stir spot welding	Al_13_Fe_4_, Al_5_Fe_2_, and Al_2_Fe
23^35^	Al alloy (surfalex 6 s) and ultrahigh strength steel	Friction stir scribe welding	Al_5_Fe_2_ (in the middle) or Al_13_Fe_4_ with Fe/Al solid solution depending on the weld regions
24^36^	Al alloy (1050) sheets and Fe particles	Friction stir processing	Al_5_Fe_2_ close to Fe particle; and Al_13_Fe_4_ close to Al matrix
25^37^	Al sheet (6061 T4) and coated steel sheet	Cold metal transfer fusion welding	Al_13_Fe_4_ (at the interface to Al) and Al_5_Fe_2_ (tongue-like, extended into steel)
26^38^	Al alloy wire (ER5356) and Zn-coated steel	Double electrode gas metal arc welding	Al_5_Fe_2_ (major) and Al_13_Fe_4_
27^39^	Pure Al and Fe	Solid Fe in liquid Al at 850 °C for 0.6 h	Al_5_Fe_2_ layer with needle-like or flake Al_13_Fe_4_
28^40^	Pure Al and Fe	Immersion testing of solid Fe and liquid Al (700–900 °C)	Al_5_Fe_2_ (adjacent to Fe) and Al_13_Fe_4_ (adjacent to Al)
29^41^	Pure Al and Fe	Immersion testing of solid Fe and liquid Al (700–900 °C)	Al_5_Fe_2_ and Al_13_Fe_4_
30^42^	Pure Al plate and pure Fe sheet	Diffusion couples	Al_5_Fe_2_ (at 873 K for 9 h) and Al_13_Fe_4_ (at 913 K for 528 h)
31^43^	Pure Al and Fe rods (diffusion couples)	Al and Fe by isothermal bonding and then annealed at 973–1073 K	Al_5_Fe_2_ (major, tongue-like) close to Fe and Al_13_Fe_4_ close to Al
32^44^	Al–Fe diffusion couples	Riveting Al rod into Fe plate	Al_13_Fe_4_ formed in Fe side at 600 °C (Al diffuses to Fe) and Al_5_Fe_2_ formed in both Al and Fe sides > 700 °C (due to Fe and Al interdiffusion)
33^46^	Hot-dip Al-coated steel	Aluminized steel at 800 °C for 60 s, then 680 °C for 60 s	Al_13_Fe_4_ just beneath Al cover layer and Al_5_Fe_2_ just underneath steel

^a^Addition of Mn promotes the formation of Al_6_(Fe,Mn).

Despite considerable observations as shown in Table 1, the underlying mechanism regarding the formation of Al–Fe IMCs is still lacking, albeit phase stability is known to be regulated by processing history involving *T* and *P* profiles for a given chemistry^47^. The phase diagram, as a foundational guide for any work in materials science and engineering^48^, is the most used tool to analyze equilibrium IMCs under a given temperature and composition (usually under external pressure *P* = 0 GPa). Additionally, non-equilibrium simulations in terms of the Scheil-Gulliver model^49,50^ can be used to analyze the forming IMCs in fast cooling processes by assuming that no diffusion takes place in the solid and that solute redistribution in the liquid is infinitely fast^51–53^. The Scheil simulations have been used, for example, to predict the formed IMCs in additively manufactured functionally graded metals^51,52^ and to predict the temperatures of liquidus and solidus in steel^53^. In addition to the phase diagram, non-equilibrium IMCs can be predicted by calculating thermodynamic driving forces for the phases of interest with respect to supercooled liquid and associated solid phases; see the predicted interface phases at the Cu/solder joints by Lee et al.^54^. Also based on thermodynamics, non-equilibrium IMCs can be tailored by partitionless solidification or by chemical partition solidification with limited atomic diffusions; for example, the non-equilibrium solidification predicted in the Al-Sm system by Zhou and Napolitano^55^. It should be remarked that thermodynamic knowledge in the literature is usually at the ambient pressure or external pressure *P* = 0 GPa, thus hindering the analysis of *P*-favored phases such as Al_6_Fe in the present work. In addition to thermodynamics, kinetics (diffusion) is another factor to regulate nucleation, growth, and coarsening of IMCs^56,57^. For example, Al_5_Fe_2_ and Al_13_Fe_4_ were formed due mainly to Al and/or Fe interdiffusion in some processes; see the examples #13, #17, #18, #30, #31, and #32 in Table 1.

The present work aims to unveil the forming mechanism of equilibrium and non-equilibrium IMCs in dissimilar aluminum to steel joints based on thermodynamic knowledge in the Al–Fe system from (1) the present first-principles and phonon calculations based on density functional theory (DFT) and (2) the CALPHAD modeling by Sundman et al.^9^ and also based on kinetic (diffusion) knowledge reported in the literature^42,58,59^. Special attention in the present DFT calculations is paid to the *P*-included Gibbs energy in addition to the variable of temperature. The challenge for the present DFT calculations is the unknown atomic configurations of (i) Al_5_Fe_2_ caused by the partially occupied Wyckoff sites 4b and 8f. of space group $Cmcm$^60^ and (ii) Al_2_Fe caused by the disordered Al and Fe in one of the Wyckoff sites 2i of space group $P\overline{1 }$^61^. To address this challenge, we adopt the following three approaches: (1) DFT-based USPEX (Universal Structure Predictor: Evolutionary Xtallography) predictions^62^, (2) DFT-based examinations of all possible configurations for a given supercell, and (3) datamining by examining all possible configurations in the literature with their formation energies predicted by machine learning. In addition to the conventional equilibrium phase diagram, non-equilibrium Scheil simulations were also used to analyze the formation of Al–Fe IMCs. The present work indicates that the forming mechanism of IMCs in dissimilar Al–Fe joints (see examples in Table 1) can be explained well using phase diagram, Scheil simulations, thermodynamic driving forces, *P*- and *T*-included Gibbs energies, and atomic diffusion coefficients in the Al–Fe system.

## Methodology

### Atomic configurations of Al–Fe IMCs

Most of the Al–Fe IMCs together with the constituent elements of FCC Al and BCC Fe are ordered structures. Their structures can be found, for example, in the Materials Project (MP) database^63^ or the Open Quantum Materials Database (OQMD)^64^; see the Supplementary Table S1. However, Al_5_Fe_2_ is an IMC with vacancies (Va) in its Wyckoff sites for Al atoms^60^. The structure of Al_5_Fe_2_ can be described by the following sublattice model according to its Wyckoff sites 4c, 8 g, 4b (occupation of 0.32 by Al), and 8f. (occupation of 0.24 by Al) of space group $Cmcm$^60^, respectively,

1 $$\left( {{\text{Fe}}} \right)_{4}^{c} \left( {{\text{Al}}} \right)_{8}^{g} \left( {{\text{Al}},{\text{Va}}} \right)_{4}^{b} \left( {{\text{Al}},{\text{Va}}} \right)_{8}^{f}$$

For another IMC of Al_2_Fe, Chumak et al.^61^ indicated that it belongs to space group $P\overline{1}$ with one of its Wyckoff sites 2i mixed with Fe (occupation of 0.705) and Al (occupation of 0.295),

2 $$\left( {{\text{Fe}}} \right)_{10} \left( {{\text{Al}}} \right)_{24} \left( {{\text{Al}},{\text{Fe}}} \right)_{4}^{i}$$

Atomic configurations of Al_5_Fe_2_ were determined as follows in the present work. First, all the independent Al_5_Fe_2_ configurations were generated by the ATAT code^65^ using a 24-atom supercell, see Eq. (1). Second, we performed DFT calculations for the 14- to 16-atom configurations with one or two Al atoms in the Wyckoff sites 4b and 8f., respectively. For the composition of Al_5_Fe_2_, we also used the universal structure predictor—USPEX^62,66^—to predict the lowest energy configuration in terms of a 14-atom supercell; where the computational engine of USPEX is DFT-based calculations ( “Details of first-principles calculations” section). In addition, we also examined the low energy configurations of Al_5_Fe_2_ suggested by Vinokur et al.^67^.

Atomic configurations of Al_2_Fe were also examined by the ATAT code^65^ based on the mixing of Al and Fe in Wyckoff site 2i (see Eq. 2) by using both the 38- and 57-atom supercells of Al_2_Fe. In addition, the MoSi_2_-type configuration suggested by Tobita et al.^68^ was included in the present work. Aiming to search for the possible configurations of Al_2_Fe, we also adopted a datamining approach by considering all the AB_2_-type configurations (~ 1.3 million) in the Materials Project (MP) database^63^, the Open Quantum Materials Database (OQMD)^64^, the Crystallography Open Database (COD)^69,70^, and the Joint Automated Repository for Various Integrated Simulations (JARVIS) database^71^. The enthalpies of formation (Δ*H*_0_) of these AB_2_-type configurations were predicted by machine learning (ML) in terms of the tool of SIPFENN (structure-informed prediction of formation energy using neural networks)^72^. Here, SIPFENN requires only atomic configurations and atomic species, which allows efficient integration into datamining study within minutes. On a random 5% subset in the OQMD structures, SIPFENN could achieve a mean absolute error of 28 meV/atom (2.7 kJ/mol-atom) to predict Δ*H*_0_^72^. For the SIPFENN suggested A_2_B-type configurations with lower Δ*H*_0_ values (more than 500 configurations were selected by considering the SIPFENN error bar up to 28 meV/atom), we performed DFT-based verifications. Notably, the present datamining approach found that the lowest energy configuration of Al_2_Fe is also the MoSi_2_-type.

### First-principles thermodynamics

Thermodynamic properties at finite temperatures can be predicted by the DFT-based quasiharmonic approach, i.e., Helmholtz energy *F* for a given phase as a function of volume *V* and temperature *T* is determined by^73,74^,

3 $$F\left( {V,T} \right) = E\left( V \right) + E_{vib} \left( {V,T} \right) + E_{el} \left( {V,T} \right) - T\left[ {S_{vib} \left( {V,T} \right) + S_{el} \left( {V,T} \right) + S_{conf} } \right]$$

Correspondingly, the Gibbs energy can be evaluated by $G\left( {P,T} \right) = F\left( {V,T} \right)\left. \right|_{P = fix} + PV$ at the given pressure of interest. Here, $E_{vib} \left( {V,T} \right)$ and $S_{vib} \left( {V,T} \right)$ are vibrational contributions (internal energy and entropy, respectively) determined by phonon densities of states (DOS’s, about 6 volumes were calculated for each phase)^73,75^. $E_{el} \left( {V,T} \right)$ and $S_{el} \left( {V,T} \right)$ are thermal electronic contributions (internal energy and entropy, respectively) determined by electronic DOS’s^73,75^. $S_{conf}$ is ideal configurational entropy introduced to account for the IMCs with partially occupied Wyckoff sites, i.e., Al_5_Fe_2_ (described by Eq. 1) and Al_2_Fe (Eq. 2),

4 $$S_{conf}^{{{\text{Al}}_{5} {\text{Fe}}_{2} }} = - \frac{{4R\left( {y_{{{\text{Al}}}}^{b} \log \left( {y_{{{\text{Al}}}}^{b} } \right) + y_{{{\text{Va}}}}^{b} \log \left( {y_{{{\text{Va}}}}^{b} } \right)} \right)}}{24} - \frac{{8R\left( {y_{{{\text{Al}}}}^{f} \log \left( {y_{{{\text{Al}}}}^{f} } \right) + y_{{{\text{Va}}}}^{f} \log \left( {y_{{{\text{Va}}}}^{f} } \right)} \right)}}{24}$$

5 $$S_{conf}^{{{\text{Al}}_{2} {\text{Fe}}}} = - \frac{{4R\left( {y_{{{\text{Al}}}}^{i} \log \left( {y_{{{\text{Al}}}}^{i} } \right) + y_{{{\text{Fe}}}}^{i} \log \left( {y_{{{\text{Fe}}}}^{i} } \right)} \right)}}{38}$$

where *R* is the gas constant and *y* the site fraction with the superscript being Wyckoff site (i.e., also the sublattice). Based on experimental measurements for Al_5_Fe_2_^60^, $y_{{{\text{Al}}}}^{b} = 0.32$ ($y_{{{\text{Al}}}}^{f} = 0.24$) and $y_{{{\text{Va}}}}^{b} = 0.68$ ($y_{{{\text{Va}}}}^{f} = 0.76$) for Al and Va, respectively. Correspondingly, $y_{{{\text{Al}}}}^{i} = 0.295$ and $y_{{{\text{Fe}}}}^{i} = 0.705$ based on experiments for Al_2_Fe^61^.

$E\left( V \right)$ in Eq. (3) is the static energy at 0 K without the zero-point vibrational energy, which was determined by fitting the DFT calculated energy-volume (*E*-*V*) data points using a four-parameter Birch-Murnaghan equation of state (EOS)^73^,

6 $$E\left( V \right) = k_{1} + k_{2} V^{ - 2/3} + k_{3} V^{ - 4/3} + k_{4} V^{ - 2}$$

where *k*_1_, *k*_2_, *k*_3_, and *k*_4_ are fitting parameters. Equilibrium properties for each phase (configuration) from this EOS include the equilibrium energy *E*_0_, volume *V*_0_, bulk modulus *B*_0_, and the pressure derivative of bulk modulus *B*′. Usually, eight reliable data points were used for each EOS fitting in the present work.

It is worth mentioning that we ignored the contribution of anharmonicity to first-principles thermodynamics in Eq. (3), which can be accounted for by using such as molecular dynamics simulations^76,77^. In the present work, the relative Gibbs energy with respect to reference states (e.g., Al and Fe) was adopted to study phase stability, making the contribution of anharmonicity cancelled to some extent. In addition, we were trying to search for the possible “low energy atomic configurations” of Al_5_Fe_2_ and Al_2_Fe (*cf.*, “Atomic configurations of Al–Fe IMCs” section), and we used the ideal configurational entropy in first-principles thermodynamics for the sake of simplicity (*cf.*, Eq. 3) for both Al_5_Fe_2_ and Al_2_Fe, although the actual configurational entropy should be considered in terms of statistical mechanics (i.e., the partition function) by including all ergodic microstates (configurations) for a system (phase) of interest^78,79^. Note that even using the lowest energy atomic configurations of Al_5_Fe_2_ and Al_2_Fe, we still need to consider configurational entropy due to the partially occupied Wyckoff sites. In summary, the sources of error in the present first-principles thermodynamics (Eq. 3) include the ignorance of anharmonicity, the adoption of ideal configurational entropy, the unknown atomic configurations of Al_5_Fe_2_ and Al_2_Fe, and the approximations used in density functional theory such as the exchange–correlation (X-C) functional^80^. Nevertheless, the DFT-based quasiharmonic approach is still a predictive tool with great success to study thermodynamics in solid phases, see the examples in our review article^47^.

### Details of first-principles calculations

All DFT-based first-principles calculations in the present work were performed by the Vienna Ab initio Simulation Package (VASP)^81^ with the ion–electron interaction described by the projector augmented wave method^82^ and the X-C functional described by the generalized gradient approximation (GGA) developed by Perdew, Burke, and Ernzerhof (PBE)^83^. The same as those in the Materials Project^63^, three electrons (3s^2^3p^1^) were treated as valence electrons for Al and fourteen (3p^6^3d^7^4s^1^) for Fe. In the VASP calculations, a plane wave cutoff energy of 293.2 eV was employed for structural relaxations and phonon calculations in terms of the Methfessel-Paxton method^84^. Final calculations of total energies and electronic DOS’s were performed by the tetrahedron method with a Blöchl correction^85^ using a wave cutoff energy of 520 eV. The employed *k*-points meshes for each structure are listed in the Supplementary Table S1. The self-consistency of total energy was converged to at least 10^−6^ eV/atom. Due to the magnetic nature of Fe, all Fe-containing materials were performed by the spin polarization calculations.

Phonon calculations were performed for each structure using the supercell approach^86^ in terms of the YPHON code^87^. Here, the VASP code was again the computational engine in calculating force constants using the finite differences method. The employed supercell for each structure and the corresponding *k*-point meshes are given in the Supplementary Table S1. In addition, the single crystal elastic constants *C*_ij_’s in the Al–Fe system were determined by applying the stress–strain method with the non-zero strains being ± 0.01; see details in^88,89^. The aggregate polycrystal properties were determined by using the Hill (H) approach^90,91^ based on the predicted *C*_ij_ values, including bulk modulus (*B*_H_), shear modulus (*G*_H_), *B*_H_/*G*_H_ ratio, Poisson’s ratio (ν_H_), and the anisotropy index *A*^U^^92^. Note that the suggested DFT settings by USPEX^62,66^ were used in the present work, aiming to search for the low energy configurations of Al_5_Fe_2_ by USPEX.

### Formation of non-equilibrium IMCs through thermodynamic analysis

The decrease in Gibbs energy, $-{\Delta G}_{m}^{\alpha }$, for the precipitation of a new phase α (e.g., IMC) from a supersaturated solution (e.g., the supercooled liquid), is the thermodynamic driving force of formation, *D*, of the new α phase, i.e., $D=-{\Delta G}_{m}^{\alpha }$^93^. The IMC with the highest thermodynamic driving force of formation can be selected as the IMC that would form first, making the driving force *D* a reasonable criterion to predict the first-forming IMC^54^. Similarly to the analysis of interface phases formed at the Cu/solder joints by Lee et al.^54^, for example, Fig. 1 shows that at 1000 K of the Al–Fe system, the supercooled liquid has a composition *x*_Fe_ = 0.163 (mole fraction of Fe in the metastable liquidus), which is in equilibrium with the supersaturated BCC phase (i.e., the metastable solidus) with *x*_Fe_ = 0.281. At this composition (*x*_Fe_ = 0.281), we can calculate thermodynamic driving forces of the IMCs (such as Al_13_Fe_4_, Al_5_Fe_2_, Al_2_Fe, and Al_8_Fe_5_) formed from the supersaturated BCC phase — the higher the driving force, the larger the possibility to form this IMC. In the present work, thermodynamic driving forces to form IMCs from the supersaturated BCC phase were calculated as a function of temperature using the modeled Al–Fe system by Sundman et al.^9^ and the Thermo-Calc software^57^.

In addition to thermodynamic driving forces, we can also use the non-equilibrium phase diagram, predicted by the Scheil-Gulliver simulations^49,50^ (see its definition in the Introduction section), to predict the formation of IMCs in fast cooling processes, such as the AM process^51,52^. Here, we used the PyCalphad software^52,94^ to calculate the Scheil non-equilibrium phase diagram with the Al–Fe thermodynamic description modelled by Sundman et al.^9^.

## Results and discussion

### DFT-based phase stability of Al–Fe IMCs

In this section, we show first the phase stability of Al–Fe IMCs at temperature *T* = 0 K and pressure *P* = 0 GPa (“DFT-based phase stability of IMCs at T = 0 K and P = 0 GPa”) aiming to demonstrate the capable of DFT-based calculations; and then, we show the phase stability of Al–Fe IMCs at finite temperatures and finite pressures (“DFT-based phase stability of IMCs at finite temperatures and finite pressures” section) through both the case studies of three reactions and the predicted *P*–*T* phase diagram.

#### *DFT-based phase stability of IMCs at T* = *0 K and P* = *0 GPa*

**Figure 3:**
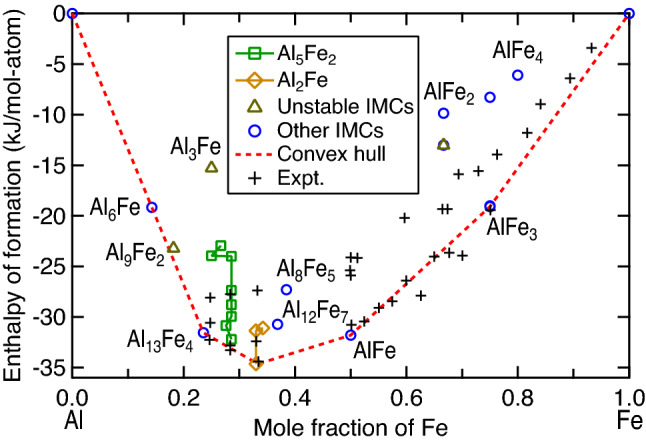
Predicted enthalpies of formation (ΔH_0_) at *T* = 0 K and *P* = 0 GPa for Al–Fe IMCs by the present DFT calculations (see structural details and ΔH_0_ values in Table S1). Note that the convex hull was plotted using the DFT results, the unstable IMCs were judged by imaginary phonon modes, and the experimental data (Expt.) were collected by Sundman et al.^9^.

**Figure 4:**
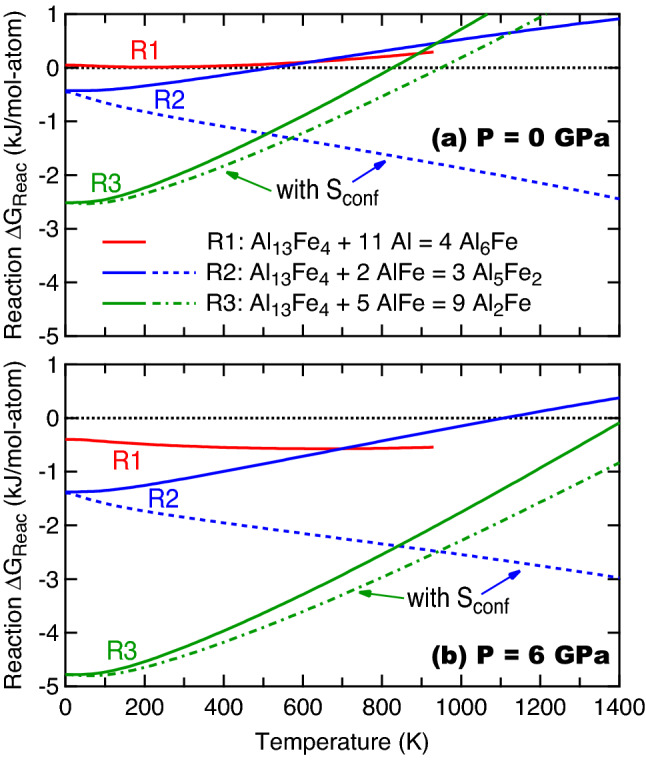
Reaction Gibbs energies (ΔG_reac_’s) under external pressure *P* = 0 GPa (**a**) and 6 GPa (**b**) with and without considering the ideal configurational entropies (S_conf_’s) of Al_5_Fe_2_ and Al_2_Fe, respectively; see Eqs. (4) and (5). The ΔG_reac_ curves for reaction R1 are plotted up to 930 K, which is slightly below the melting point of Al (933 K).

Figure 3 shows the predicted values of enthalpy of formation (ΔH_0_) for Al–Fe IMCs based on the present DFT calculations at *T* = 0 K and *P* = 0 GPa. Detailed atomic configuration and ΔH_0_ value of each IMC are given in the Supplementary Table S1; in particular, the predicted 14-atom configuration of Al_5_Fe_2_ by USPEX^62,66^ is listed in the Supplementary Table S2. Figure 3 shows also the convex hull by DFT calculations to display the stable IMCs, the experimental ΔH_0_ values collected by Sundman et al.^9^ to measure the quality of the present DFT calculations, and the unstable configurations judged by imaginary phonon modes (not shown). It can be seen that (i) the DFT-predicted ΔH_0_ values agree well with the experimental data which are scattered; (ii) Al_6_Fe is close to but above the convex hull, indicating that it is metastable at *T* = 0 K and *P* = 0 GPa, and more attentions need to be paid to its phase stability at high temperatures and high pressures; (iii) Al_9_Fe_2_ is an unstable structure due to the existence of imaginary phonon modes and hence ignored in the present work; (iv) Al_5_Fe_2_ is a metastable phase at *T* = 0 K and *P* = 0 GPa, although various configurations have been examined in the present work (see the green open squares as well as the details in Table S1); (v) the MoSi_2_-type Al_2_Fe possesses the lowest energy and on the convex hull at *T* = 0 K and *P* = 0 GPa, but this configuration doesn’t belong to space group $P\overline{1 }$ as suggested by Chumak et al.^61^; and (vi) the IMCs of Al_13_Fe_4_, AlFe, and AlFe_3_ are stable based on the convex hull. Figure 3 implies that, at the conditions of *T* = 0 K and *P* = 0 GPa, the DFT predicted ΔH_0_ values for Al_5_Fe_2_ and non-MoSi_2_-type Al_2_Fe (i.e., the triclinic Al_2_Fe^61^) are close to but above the convex hull, indicating that (a) the supercells used in the present work may be too small to search for the lowest energy atomic configurations, and (b) additional effects on phase stability such as temperature, pressure, and new approaches need to be considered. To this end as well as the suggestions by Fig. 3, phase stabilities of Al_6_Fe, Al_5_Fe_2_, and Al_2_Fe are further examined at finite temperatures and finite pressures (see Fig. 4).

#### DFT-based phase stability of IMCs at finite temperatures and finite pressures

Phase diagram at a given temperature and pressure can be constructed using the convex hull approach, i.e., by examining all reaction Gibbs energies, $\Delta {G}_{\mathrm{reac}}$, for a system of interest. Note that in general one reaction cannot determine phase stability in the whole temperature and pressure ranges. As test cases, Fig. 4 shows only the changes of $\Delta G_{{{\text{reac}}}}$ as a function of temperature and pressure (*P* = 0 and 6 GPa as two examples) for the following three reactions, aiming to understand phase stability of Al_6_Fe as well as Al_5_Fe_2_ and Al_2_Fe with respect to the given reference phases (instead of building the convex hull),

7 $${\text{R}}1:{\text{ Al}}_{13} {\text{Fe}}_{4} + 11 {\text{Al}} = 4 {\text{Al}}_{6} {\text{Fe}}$$

8 $${\text{R}}2:{\text{ Al}}_{13} {\text{Fe}}_{4} + 2 {\text{AlFe}} = 3 {\text{Al}}_{5} {\text{Fe}}_{2}$$

9 $${\text{R}}3:{\text{ Al}}_{13} {\text{Fe}}_{4} + 5 {\text{AlFe}} = 9 {\text{Al}}_{2} {\text{Fe}}$$

Here we choose the stable phases of Al, Al_13_Fe_4_, and AlFe (the B2 structure) as the reference states to examine phase stability of Al_6_Fe, Al_5_Fe_2_ (using the configuration predicted by USPEX), and Al_2_Fe (using the MoSi_2_-type configuration predicted by SIPFENN). As mentioned at the end of “First-principles thermodynamics” section, the ideal configurational entropies together with the possible “low energy configurations” were used for Al_5_Fe_2_ and Al_2_Fe, resulting in a large contribution of configurational entropy than the actual case. However, the predicted configurations of Al_5_Fe_2_ and Al_2_Fe are still not the lowest energy ones based on the present approach, making the error by using the larger ideal configurational entropy cancelled to some extent. Also the $\Delta {G}_{\mathrm{reac}}$ values with and without the contributions of ideal configurational entropy form an uncertainty range to analyze phase stability of Al–Fe IMCs.

Figure 4 shows that Al_6_Fe is a *T-*unfavored (see R1) but a *P-*favored phase, which can be understood through phonon density of states as detailed in Supplementary Materials. Figure 4b. It shows that with increasing pressure (even less than 1 GPa) instead of increasing temperature, Al_6_Fe becomes stable with respect to Al and Al_13_Fe_4_ (*cf.*, the reaction R1). Based on experimental observations such as the examples #1 to #5 in Table 1, Al_6_Fe was formed in the processes associated with pressures (such as die casting and equal channel angular extrusion) and in high Al-containing samples (e.g., *x*_Al_ > 0.9). The reaction R2 (see Eq. 8) in Fig. 4a and b shows that Al_5_Fe_2_ is a *T*-unfavored but *P*-favored phase by ignoring the contribution of configurational entropy *S*_conf_; see Eqs. 3 and 4. With *S*_conf_ contribution to $\Delta {G}_{\mathrm{reac}}$, Al_5_Fe_2_ becomes both the *T*- and *P*-favored phase (see the blue dash lines of R2). These results indicate that factors including atomic configuration, temperature, pressure, and *S*_conf_ make Al_5_Fe_2_ more stable. Figure 4 also shows that the MoSi_2_-type Al_2_Fe is *T*-unfavored, but it is a strong *P*-favored phase. In addition, the *S*_conf_ has less contribution to $\Delta {G}_{\mathrm{reac}}$ in comparison with that for Al_5_Fe_2_, due to the less partially occupied Wyckoff site of Al_2_Fe; see Eqs. (4) and (5). The *T*-unfavored behavior is caused by the lower phonon DOS of Al_2_Fe than those of AlFe and Al_13_Fe_4_; see details in Supplementary Material. With increasing pressure, Fig. 4 shows that the $\Delta {G}_{\mathrm{reac}}$ value of reaction R3 decreases greatly; for example, dropping more than 2 kJ/mol-atom at *T* = 0 K as well as at other temperatures. Experimentally, the MoSi_2_-type Al_2_Fe was synthesized through the laser-heated diamond-anvil cell at 10 GPa and 1873 K^95^, and it was suggested that it is a high pressure phase existing at *P* > 5 GPa^68^; these experiments agree with the present conclusion that Al_2_Fe is a *T*-unfavored but a strong *P*-favored phase, albeit it is stable at *T* = 0 K and *P* = 0 GPa (Fig. 3).

**Figure 5:**
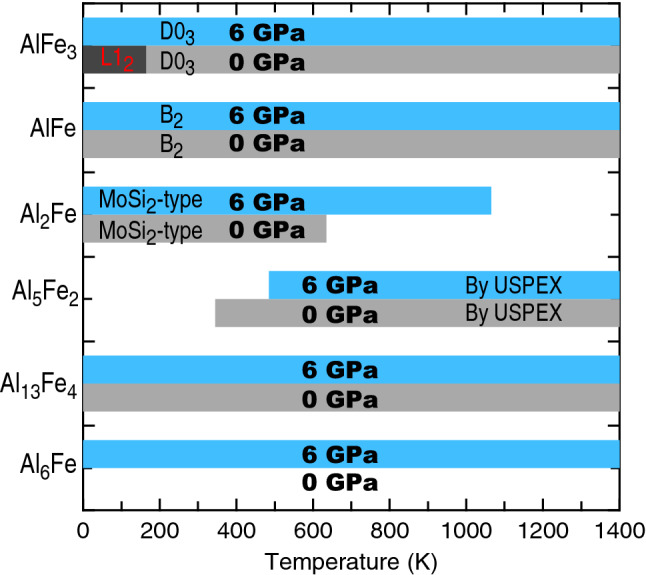
Phase stability (on the convex hull) of Al–Fe IMCs under external pressures of 0 and 6 GPa as a function of temperature (shown in the shaded regions) based on the present DFT calculations using Eq. (3). The predicted L1_2_-AlFe_3_ presented at low temperatures is due to the employed X-C functional of GGA, see discussion in main text.

Figure 5 shows a schematic *P*–*T* phase diagram (demonstrated with *P* = 0 and 6 GPa) for the Al–Fe system based on the present DFT calculations using Eq. (3) based on the convex hull approach by considering all $\Delta {G}_{\mathrm{reac}}$ values. As an example, the $\Delta {G}_{\mathrm{reac}}$ values at *P* = 0 GPa for six reactions are shown in the Supplementary Figure S2, where the reaction R4 can be used to determine the critical temperatures of Al_5_Fe_2_ in some temperatures and pressures. Figure 5 indicates that Al_13_Fe_4_, AlFe, and AlFe_3_ are always the stable IMCs marked by the shaded regions. However, at low pressures and low temperatures (e.g., *P* = 0 GPa and *T* < 165 K), the L1_2_-type AlFe_3_ is more stable than the D0_3_-type AlFe_3_. It is worth mentioning that AlFe_3_ from DFT-based predictions is either a L1_2_ structure or a D0_3_ structure depended on the selected X-C functional^96,97^. The commonly used X-C functional of GGA predicts that the L1_2_-AlFe_3_ is more stable at 0 K with respect to the D0_3_-AlFe_3_ (see Table S1 as well as the results in the literature^63,96,97^). However, the energy difference between the L1_2_ and D0_3_ structures of AlFe_3_ is very small (< 0.1 kJ/mol-atom, see Table S1), which is within the uncertainty of DFT predictions. Regardless of the stable structure at 0 K for AlFe_3_ (L1_2_ vs. D0_3_), the present work shows that vibrational entropy makes the D0_3_ structure more stable at high temperatures (> 165 K and *P* = 0 GPa) and/or at high pressures (> ~ 1 GPa); agreeing with the experimentally observed AlFe_3_ with the D0_3_ structure^9^. Over the entire temperature range in Fig. 5, Al_6_Fe is not stable at *P* = 0 GPa, but is stable at higher pressures. Al_5_Fe_2_ (configuration predicted by USPEX) is stable at high temperatures (e.g., *T* > 345 K with *P* = 0 GPa), while higher pressures decrease its stability slightly. The MoSi_2_-type Al_2_Fe is a *T*-unfavored but a strong *P*-favored phase.

In comparison with the IMCs reported in the CALPHAD modelled Al–Fe phase diagram at *P* = 0 K and low temperatures (e.g., < 1000 K in Fig. 1), the DFT-based predictions in Fig. 5 agree reasonably well with those by the CALPHAD modeling, including the existed Al_13_Fe_4_, AlFe, and AlFe_3_, as well as the absent Al_6_Fe. The deviations are only for Al_5_Fe_2_ and Al_2_Fe, which are stable at low temperatures (e.g., < 1000 K) by CALPHAD modeling but are not always stable by DFT-based predictions, indicating that the present configurations of Al_5_Fe_2_ and Al_2_Fe are still not the lowest energy ones. It should be mentioned that the presently predicted Al_5_Fe_2_ configuration (see Table S2) has the lowest energy than the configurations reported in the literature (see Table S1)^67^, while the presently predicted MoSi_2_-type Al_2_Fe is the same as the one suggested by Tobita et al.^68^ To the best of our knowledge, the present configurations are the lowest energy ones which can be currently predicted for Al_5_Fe_2_ and Al_2_Fe, but future efforts are still needed to predict new lower energy configurations by using larger supercells or new approaches.

Table 2 summarizes phase stability of Al–Fe IMCs as a function of pressure and temperature as shown in Figs. 1, 3, and 5; together with their ductility/brittleness judged by Pugh’s criterion^11,12^ as shown in Fig. 2, which were determined by the presently predicted elastic constants in Table S3.

**Table 2:** Summary of phase stability of key Al–Fe IMCs with respect to pressure (*P*) and temperature (*T*) shown in Figs. 1, 3, and 5 (or not shown); together with their ductility/brittleness according to Pugh’s criterion^11,12^ as shown in Fig. 2.

Al–Fe IMCs^a^	Ductility	*P*-favored?	*T*-favored?
Al_6_Fe	Ductile	Yes	Not, or less effect
Al_13_Fe_4_ (θ, Al_3_Fe^9^)	Slightly brittle	Always on convex hull^b^	Always on convex hull^b^
Al_5_Fe_2_ (η, Al_8_Fe_3_^98^)	Brittle	Not, or less effect	Yes
Al_2_Fe (MoSi_2_-type)	Brittle	Yes, and strong	Not
Al_8_Fe_5_ (D8_2_, ε^9^)	Ductile	Less effect	Less effect
AlFe (B_2_)	Slightly brittle	Always on convex hull^b^	Always on convex hull^b^
AlFe_3_ (D0_3_)	Ductile	Yes	Yes

^a^Names used in the present work together with the names in the parentheses used in the literature.

^b^These IMCs are always stable and on the convex hull in the present *P* and *T* of studied.

### Non-equilibrium Al–Fe IMCs by thermodynamic and kinetic analyses

In this section, we show first the formation of non-equilibrium IMCs by thermodynamic driving forces and kinetic analyses (“Non-equilibrium IMCs by thermodynamic driving forces and kinetic analyses” section); and then, we show the formation of non-equilibrium IMCs by Scheil simulations (“Non-equilibrium IMCs by Scheil simulations” section).

#### Non-equilibrium IMCs by thermodynamic driving forces and kinetic analyses

**Figure 6:**
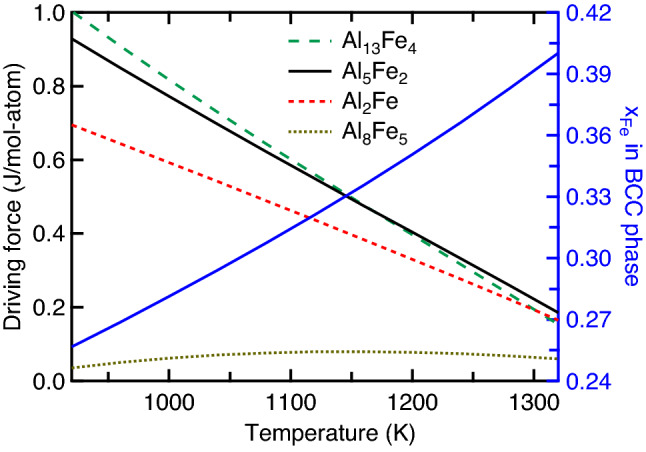
Thermodynamic driving forces of Al–Fe IMCs as a function of temperature (*T* = 920–1320 K) as well as the associated mole fraction of Fe (*x*_Fe_ = 0.26–0.40) along the metastable solidus line as shown in Fig. 1. Here, the Al–Fe thermodynamic properties were modelled by Sundman et al.^9^.

**Figure 7:**
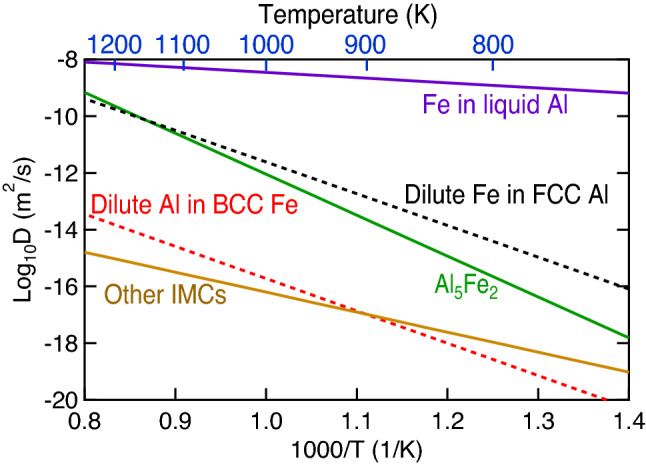
Diffusion coefficients of Fe in liquid Al^58^, dilute Fe in FCC Al^58^, dilute Al in BCC Fe^59^, and the elements in Al_5_Fe_2_ and other IMCs (AlFe, Al_2_Fe, and Al_13_Fe_4_)^42^.

Figure 6 shows the predicted thermodynamic driving forces of the Al–Fe IMCs as a function of temperature (*T* = 920–1320 K) as well as the associated mole fraction of Fe (*x*_Fe_ = 0.28–0.40) along the metastable solidus line as shown in Fig. 1. Note that the eutectic reaction temperature is 927 K and the chosen thermodynamic description was that modelled by Sundman et al.^9^. It is seen that both Al_13_Fe_4_ and Al_5_Fe_2_ have the higher thermodynamic driving forces of formation at lower temperatures (< 1280 K) than those of Al_2_Fe and Al_8_Fe_5_. By examining atomic diffusivity in Al–Fe IMCs, the interdiffusion coefficients in Al_5_Fe_2_ are at least two orders of magnitude faster than those in the other IMCs (AlFe, Al_2_Fe, and Al_13_Fe_4_) at *T* = 823 – 913 K^42^ and are comparable with the diffusion coefficients of dilute Fe in FCC Al; see Fig. 7 the diffusion coefficients reported in the literature^42,58,59^. In addition, Al atoms have higher diffusivity in Al_5_Fe_2_ than Fe atoms^38^. The fastest atomic diffusivity, especially Al atoms, in Al_5_Fe_2_ is due mainly to the rich Al vacancies in Al_5_Fe_2_^60^; see Eq. (1). However, considerable vacancies have not been reported in the other Al–Fe IMCs. By considering both the high thermodynamic driving force of formation (Fig. 6) and the fastest interdiffusion coefficients (Fig. 7), the brittle Al_5_Fe_2_ is the IMC with the largest possibility to be formed; see the Al-rich examples in Table 1, excepting those with extremely high Al contents, formed below the eutectic reaction temperature of 927 K, or processed by AM (examples #1 to #5, and #7 to #11).

#### Non-equilibrium IMCs by Scheil simulations

**Figure 8:**
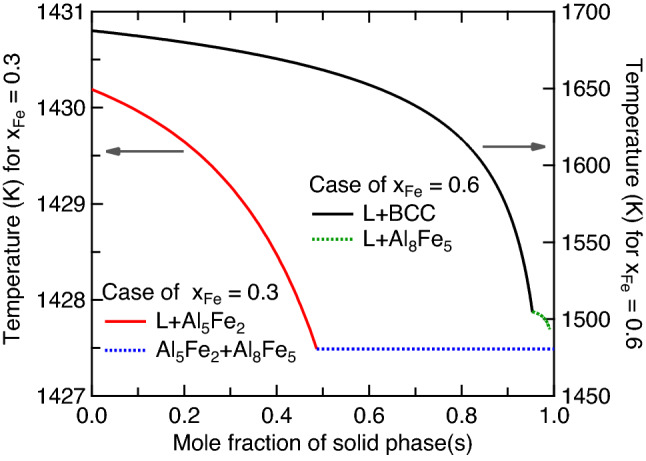
Calculated mole fractions of solid phases with *x*_Fe_ = 0.3 and 0.6 based on Scheil simulations using the thermodynamic description modelled by Sundman et al.^9^.

**Figure 9:**
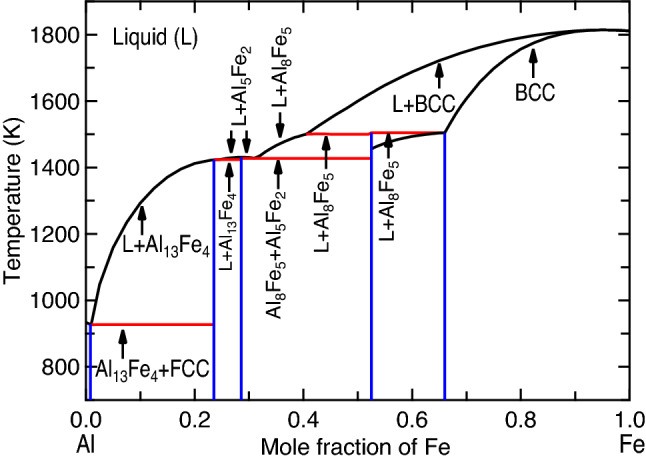
Predicted Al–Fe non-equilibrium phase diagram by Scheil simulations uisng the modelled data by Sundman et al.^9^, showing the forming temperatures for the phases indicated by the lines. Note that the lever rule cannot be used to determine phase fractions (see Fig. 8 for two examples).

As two examples of fast cooling solidification, Fig. 8 shows the calculated mole fractions of solid phases by Scheil simulations using the thermodynamic description modelled by Sundman et al.^9^. With decreasing tempeature at the fixed composition of *x*_Fe_ = 0.3, the solid phase of Al_5_Fe_2_ forms first and reaches a maximum mole fracition about 0.5 at *T* = 1427.5 K, and then the second solid phase of Al_8_Fe_5_ forms at almost the fixed temperature of 1427.5 K. Due to the exteremely small temperture range (< < 1 K) for phase transition, Al_8_Fe_5_ was not observed in all the processes in Table 1. For the case of *x*_Fe_ = 0.6, the first solid phase formed with decreasing temperature is BCC (or the B2 phase), which reaches a maximum mole fraction of 0.95, and then Al_8_Fe_5_ forms in a small temperature range of 1505–1493 K. Similar to the case of *x*_Fe_ = 0.3, the predicted Al_8_Fe_5_ was also not observed in the processes in Table 1 due probably to the small temperature range of phase formation. Figure 9 shows the complete non-equilibrium phase diagram by Scheil simulations using the modelled Al–Fe system by Sundman et al.^9^. This non-equilibrium phase diagram shows the temperatures of the forming phases, though the lever rule cannot be used to determine phase fractions. Both the equilibrium phase diagram (Fig. 1) and the Scheil non-equilibrium phase diagram (Fig. 9) can be used to determine the forming phases in the slow/equilibrium and the fast cooling processes, respectively.

**Figure 10:**
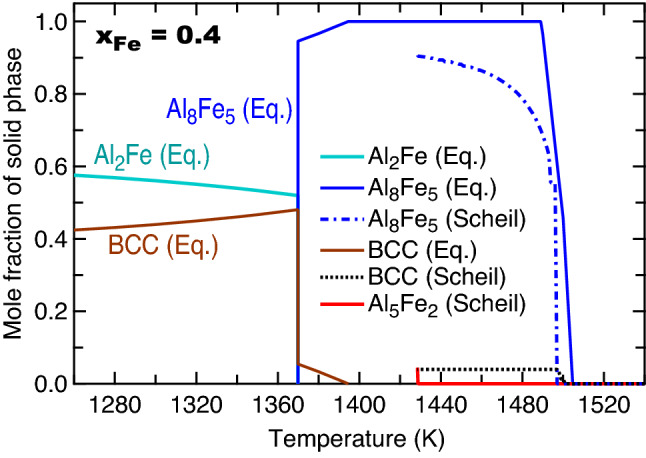
Calculated mole fracitons of solid phases with *x*_Fe_ = 0.4 based on Scheil simulations and equilibrium (Eq.) calculations using the modelled data by Sundman et al.^9^.

As an example to examine equilibrium and Scheil simulations, Fig. 10 show the forming phases as a function of temperature with *x*_Fe_ = 0.4. The forming phases are BCC and Al_8_Fe_5_ (majorly) based on Scheil simulations (see also Fig. 9), while the forming phases are Al_8_Fe_5_ (when *T* > 1360 K), BCC, and Al_2_Fe based on equilibrium calculations (see also Fig. 1). Therefore, the forming phases could be BCC, Al_2_Fe, and/or Al_8_Fe_5_ depended on different processes. For instance, Stein et al.^99^ observed the eutectoid reaction of Al_8_Fe_5_ ↔ Al_2_Fe + BCC (B2) at 1368 K by the differential thermal analyses for the Al-40 at.% Fe alloy (*x*_Fe_ = 0.4) at the heating rates of 5 and 10 K/min.

It should be remarked that the forming phases depend mainly on compositions (especially the local compositions) in addition to temperature, pressure, and atomic diffusivity for the system of interest. Table 1 shows that AM is superior to the other processes in achieving desired phases such as AlFe and AlFe_3_ through varying the compositions. To predict the forming IMCs under a given composition and a given processing history, the combined thermodynamic and kinetic simulations are needed. For example, Lindwall et al.^100^ simulated the time–temperature-transformation (TTT) diagram and the forming IMCs in the additively manufactured Ni-based Inconel 625. However, these simulations are beyond the scope of the present work.

## Summary

The present work investigated the forming mechanism of equilibrium and non-equilibrium intermetallic compounds (IMCs) in dissimilar aluminum/steel (Al–Fe) joints by means of Gibbs energy as a function of temperature (*T*) and pressure (*P*) from (i) first-principles phonon calculations, (ii) equilibrium Al–Fe phase diagram in the literature and the presently predicted non-equilibrium phase diagram by Scheil simulations, (iii) atomic diffusivity in Al–Fe, and (iv) experimentally observed IMCs in various processes (*cf.*, Table 1). In particular, the unknown atomic configurations of Al_2_Fe and Al_5_Fe_2_ were examined in the present work by machine learning based datamining together with first-principles verifications and structure predictor (using USPEX). To the best of our knowledge, the presently predicted configurations of Al_2_Fe and Al_5_Fe_2_ possess lower energies in comparison with the configurations reported in the literature. However, the present configurations are still not the lowest energy ones, hence appealing for future efforts. In addition, the predicted MoSi_2_-type Al_2_Fe is a pressure-favored IMC, instead of the phase with space group $P\overline{1 }$ shown in the experimental phase diagram. Note that the present DFT-based thermodynamics is based on the quasiharmonic approach with the possible sources of error from such as the ignorance of anharmonicity, the adoption of ideal configurational entropy, the unknown atomic configurations of Al_5_Fe_2_ and Al_2_Fe, and the approximations adopted in density functional theory.

Al–Fe IMCs formed in various experimental processes are summarized in Table 1 (“Introduction” section). The present work concludes that the formation of IMCs can be explained well by phase diagrams, thermodynamic driving forces, *P*- and *T*-included Gibbs energy, and atomic diffusion coefficients. Specifically, the metastable and ductile Al_6_Fe is a *P*-favored IMC, which was observed in Al-dominant samples and the processes involving pressures such as direct-chill casting, die casting, equal channel angular extrusion. Here the ductility and brittleness of IMCs were judged by Pugh’s criterion^11,12^ using the presently predicted elastic constants. The MoSi_2_-type Al_2_Fe is a brittle and strong *P*-favored IMC observed at high pressures. The stable but brittle η-Al_5_Fe_2_ is the most observed IMC usually adjacent to steel (Fe) in almost all the processes as detailed in Table 1, such as fusion or solid-state welding, immersion testing, diffusion couples, and additive manufacturing (AM), since Al_5_Fe_2_ is a *T*-favored phase with a high thermodynamic driving force of formation and the fastest atomic diffusivity among all Al–Fe IMCs. The slightly brittle θ-Al_13_Fe_4_ is the second most observed IMC usually adjacent to Al shown in most of the processes, possessing the highest thermodynamic driving force of formation in Al-rich side. Notably, the ductile AlFe_3_, the less ductile AlFe, and almost all the other IMCs were observed in the AM processes, making AM an exceptional way to tailor composition and in turn achieve the desired IMCs in dissimilar materials. All the IMCs (without the *P*-favored phases) formed in the Al–Fe joints can be identified using the equilibrium and the Scheil non-equilibrium phase diagrams, together with kinetic considerations.

## Supplementary Information

Supplementary Information.
