# Supplementary material for: Forming mechanism of equilibrium and non-equilibrium metallurgical phases in dissimilar aluminum/steel (Al–Fe) joints
Source: Sci Rep. 2021 Dec 20;11:24251. doi: 10.1038/s41598-021-03578-0 (PMC8688452; doi:10.1038/s41598-021-03578-0)
Supplement: Supplementary file 1 — Supplementary Information. [file 41598_2021_3578_MOESM1_ESM.docx]

**Supplementary Materials**

**Forming mechanism of equilibrium and non-equilibrium metallurgical phases in dissimilar aluminum/steel (Al-Fe) joints**

Shun-Li Shang^a,*^, Hui Sun^a^, Bo Pan^b^, Adam M. Krajewski^a^, Yi Wang^a^, Mihaela Banu,^c^

Jingjing Li^b^, Zi-Kui Liu^a^

^a^ Department of Materials Science and Engineering, Pennsylvania State University, University Park, PA 16802, USA

^b^ Department of Industrial and Manufacturing Engineering, Pennsylvania State University, University Park, PA 16802, USA

^c^ Department of Mechanical Engineering, University of Michigan, Ann Arbor, MI 48109, USA

*E-mail: sus26@psu.edu

**Understanding phase stability from phonon density of states (DOS)**

Phase stability of Al_6_Fe (see Figure 4, as well as other IMCs) at finite temperatures and under a given pressure can be understood by vibrational entropy, $S_{vib}$, in terms of the phonon density of states (DOS), $g(\omega)$ ^1^,

| $S_{vib}\propto\int g\left( \omega\right)ln(\omega)d\omega$ | Eq. S1 |
| --- | --- |

where $\omega$ is phonon frequency. Eq. S1 indicates that the higher the phonon DOS in the low $\omega$ region, the higher the $S_{vib}$ contribution to Gibbs energy will be (see Eq. 3) ^1–3^. Figure S1 shows the phonon DOS’s of FCC Al and the selected Al-Fe IMCs at *P* = 0 GPa. Note that the shape of phonon DOS for each phase is similar at *P* = 0 GPa and high pressures. In general, it shows that the $g(\omega)$ of Al has a higher density than the Al-Fe IMCs in the low frequency region (e.g., $\omega$ < 6 THz), since FCC Al is the softest material with the largest equilibrium volume *V*_0_ and the smallest bulk modulus *B*_0_ in the Al-Fe system; see the Supplementary Table S 1. Relevant to the reaction R1 (Eq. 7) and at the low frequency region ($\omega$ < 6 THz), Figure S1 shows that the $g(\omega)$ of Al is much higher than that of Al_6_Fe with $3.5<\omega<6$ THz, but the $g(\omega)$ of Al_6_Fe has higher values with $\omega<2$ THz. In addition, the phonon DOS’s of Al and Al_13_Fe_4_ do not have significant differences at the low frequency region. These features imply that the $S_{vib}$ contributions to both Al and Al_13_Fe_4_ should be slightly higher than that of Al_6_Fe, resulting a slight increase of $\Delta G_{\mathrm{reac}}$ for reaction R1 with increasing temperature (at *P* = 0 GPa). At higher pressures such as *P* = 6 GPa, $\Delta G_{\mathrm{reac}}$ for reaction R1 remains roughly constant since the $S_{vib}$ contributions to both Al and Al_13_Fe_4_ are nearly identical to that of Al_6_Fe.


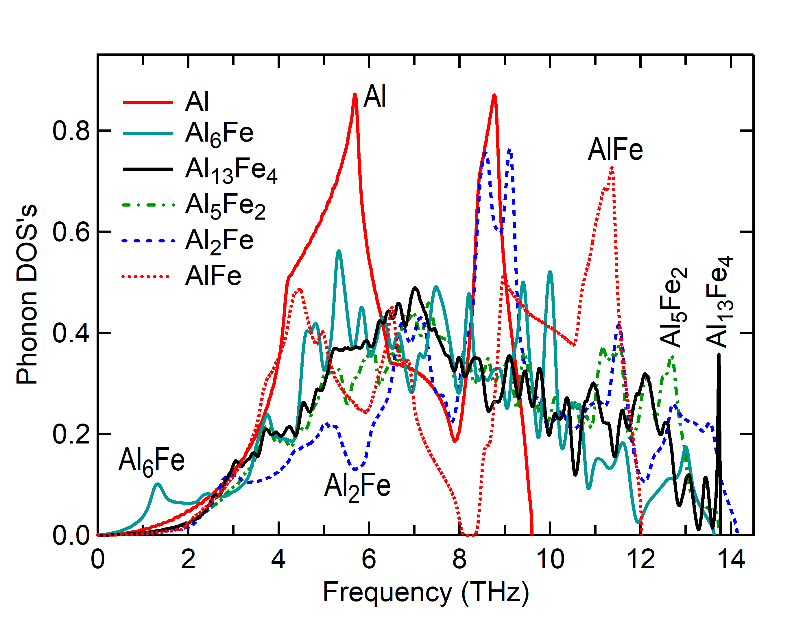


**Figure S 1.** Calculated phonon densities of states (DOS’s) of fcc Al and the selected IMCs of Al_6_Fe, Al_13_Fe_4_, Al_5_Fe_2_ (predicted by USPEX), the MoSi_2_-type Al_2_Fe, and AlFe (the B2 phase). Note that all phonon DOS’s have the same integral value.


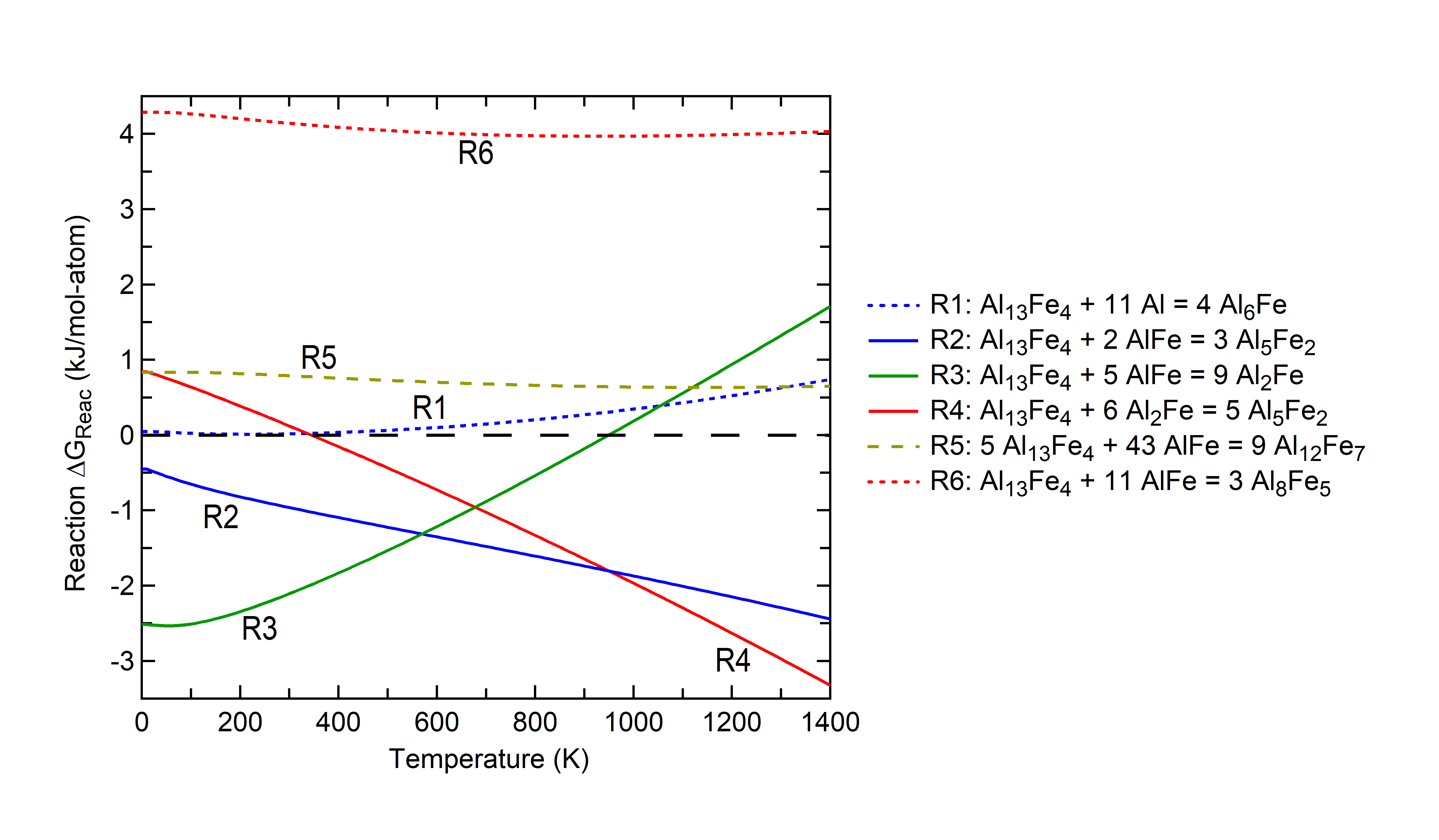


**Figure S 2.** Six reaction Gibbs energies (ΔG_reac_’s) under external pressure *P* = 0 GPa by considering the ideal configurational entropies (S_conf_’s) of Al_5_Fe_2_ and Al_2_Fe, respectively; see Eq. 4 and Eq. 5.

Table S 1. Details of DFT calculations together with the predicted equilibrium properties (*V*_0_, *B*_0_, *B’*, and ΔH_0_ with respect to FCC Al and BCC Fe) by Eq. 6. Experimental data in the literature (except ΔH_0_, *cf.* Figure 3) are also shown when available. The “structures” refer to those used in the Materials Project (mp) database ^4^, this work, etc. *x*_Fe_ indicates mole fraction of Fe; *C*_ij_ and TC indicate elastic constants and thermochemical properties by Eq. 3, respectively, predicted in the present work (Yes, Y) or not (empty). Atoms and numbers in the parentheses indicate atoms in the supercells and *k*-point meshes used in the DFT, *C*_ij_, and phonon calculations. Spin MM indicates the spin moment at the equilibrium volume.

| Name | Structures | Space  group | *x*_Fe_ | *C*_ij_ | TC | Atoms (*k*-mesh): DFT/*C*_ij_ | Atoms (*k*-mesh): Phonon | Spin MM  (μ_B_/atom) | *V_0_*  (Å^3^/atom) | *B*_0_  (GPa) | *B’* | ΔH_0_ in  kJ/mol-atom |
| --- | --- | --- | --- | --- | --- | --- | --- | --- | --- | --- | --- | --- |
| FCC Al | mp-134 | $Fm\bar{3}m$ | 0.000 | Y | Y | 4 (27×27×27) | 32 (7×7×7) | 0.00 | 16.496  16.58^a^ | 77.8  79.4^a^ | 4.43 | 0.00 |
| Al_6_Fe | mp-570001 | $Cmcm$ | 0.143 | Y | Y | 28 (5×6×5) | 28 (5×6×5) | 0.00 | 15.073  15.16^b^ | 105.2 | 4.38 | -19.17 |
| Al_9_Fe_2_ | mp-1191778 | $P2_{1}/c$ | 0.182 |  |  | 22 (7×6×5) | 88 (2×2×3) | 0.00 | 15.353 | 93.6 | 5.49 | -23.23 |
| Al_13_Fe_4_ | Ref. ^5^ | $C2/m$ | 0.235 | Y | Y | 102 (1×2×2) | 102 (1×2×2) | 0.00 | 14.389  14.60^c^ | 121.4 | 4.40 | -31.55 |
| Al_3_Fe | mp-984873 | $P6_{3}/mmc$ | 0.250 |  | Y | 8 (13×13×13) | 64 (4×4×4) | 0.10 | 13.658 | 125.1 | 4.48 | -15.28 |
| Al_5_Fe_2_ (1) ^d^ | This work | $Cmcm$ | 0.250 |  | Y | 16 (8×6×5) | 64 (3×2×4) | 0.03 | 14.004 | 120.4 | 4.81 | -23.95 |
| Al_5_Fe_2_ (2) ^d^ | This work | $Cmcm$ | 0.250 |  | Y | 16 (8×6×5) | 64 (3×2×4) | 0.03 | 14.005 | 120.3 | 4.73 | -23.95 |
| Al_5_Fe_2_ (3) ^d^ | This work | $Cmcm$ | 0.267 |  | Y | 15 (12×8×6) | 60 (3×2×4) | 0.04 | 14.261 | 119.2 | 4.57 | -22.95 |
| Al_5_Fe_2_ (4) ^d^ | This work | $Cmcm$ | 0.267 |  | Y | 15 (12×8×6) | 60 (3×2×4) | 0.05 | 14.260 | 119.3 | 4.61 | -22.95 |
| Al_5_Fe_2_ (5) ^d^ | This work | $Cmcm$ | 0.286 | Y | Y | 14 (10×6×5) | 56 (4×2×4) | 0.00 | 13.963 | 126.8 | 4.30 | -29.95 |
| Al_5_Fe_2_ (6) ^e^ | By USPEX | $C2/m$ | 0.286 | Y | Y | 14 (11×6×5) | 56 (4×2×4) | 0.00 | 13.626  13.63 ^f^ | 132.4 | 4.23 | -32.22 |
| Al_5_Fe_2_ (m1) ^g^ | Model1 ^6^ | $Cmcm$ | 0.276 |  |  | 29 (5×6×5) |  | 0.00 | 13.948 | 125.4 | 4.47 | -30.85 |
| Al_5_Fe_2_ (m2) ^g^ | Model2 ^6^ | $Cmcm$ | 0.286 |  |  | 28 (5×6×5) |  | 0.00 | 14.426 | 120.1 | 4.25 | -28.80 |
| Al_5_Fe_2_ (m3) ^g^ | Model3 ^6^ | $Cmcm$ | 0.286 |  |  | 14 (7×8×12) |  | 0.00 | 14.298 | 122.4 | 4.60 | -23.99 |
| Al_5_Fe_2_ (m5) ^g^ | Model5 ^6^ | $Cmcm$ | 0.286 |  |  | 28 (5×6×5) |  | 0.00 | 14.360 | 122.9 | 4.24 | -27.38 |
| Al_2_Fe (MoSi_2_) | MoSi_2_-type  (mp-2592) | $I4/mmm$ | 0.333 | Y | Y | 6 (20×20×7) | 54 (5×5×5) | 0.00 | 12.803  13.01^h^ | 147.2 | 4.42 | -34.63 |
| Al_2_Fe (1)^i^ | This work | $P\bar{1}$ | 0.333 | Y | Y | 57 (4×3×2) | 57 (4×3×2) | 0.27 | 13.620  13.83^j^ | 125.6 | 4.82 | -31.36 |
| Al_2_Fe (2) ^i^ | This work | $P\bar{1}$ | 0.342 | Y | Y | 38 (5×4×3) | 38 (5×4×3) | 0.29 | 13.578  13.83^j^ | 125.6 | 5.11 | -31.09 |
| Al_12_Fe_7_ | mp-1214901 | $P\bar{1}$ | 0.368 |  | Y | 19 (8×6×4) | 76 (2×2×2) | 0.44 | 13.531 | 123.8 | 4.14 | -30.72 |
| Al_8_Fe_5_ (D8_2_) | mp-1193259 | $I\bar{4}3m$ | 0.385 | Y | Y | 52 (5×5×5) | 52 (5×5×5) | 0.59 | 13.183  13.906^k^ | 138.0 | 4.14 | -27.30 |
| AlFe (B2) | mp-2658 | $Pm\bar{3}m$ | 0.500 | Y | Y | 2 (31×31×31) | 54 (4×4×4) | 0.35 | 11.896  12.317^k^ | 174.3 | 4.43 | -31.79 |
| AlFe (Hex) | mp-985578 | $P6/mmm$ | 0.500 |  | Y | 2 (28×28×13) | 36 (5×5×4) | 1.21 | 14.938 | 90.7 | 4.59 | 45.17 |
| AlFe_2_ (Hex) | mp-985579 | $P6/mmm$ | 0.667 |  | Y | 3 (17×17×24) | 36 (5×5×5) | 1.48 | 12.654 | 143.9 | 3.56 | -13.03 |
| AlFe_2_ (Cubic) | mp-31184 | $Fd\bar{3}m$ | 0.667 |  | Y | 24 (7×7×7) | 192 (1×1×1) | 1.10 | 12.559 | 129.6 | 5.26 | -9.85 |
| AlFe_3_ (L1_2_) | mp-1183162 | $Pm\bar{3}m$ | 0.750 | Y | Y | 4 (21×21×21) | 108 (2×2×2) | 1.72 | 12.206 | 169.1 | 3.89 | -19.10 |
| AlFe_3_ (D0_3_) | mp-2018 | $Fm\bar{3}m$ | 0.750 | Y | Y | 16 (9×9×9) | 128 (1×1×1) | 1.48 | 11.864  12.14^m^ | 164.1 | 5.74 | -19.02 |
| AlFe_3_ (Orth) | mp-1228919 | $Cmmm$ | 0.750 |  | Y | 8 (16×12×6) | 48 (3×3×3) | 1.60 | 12.139 | 149.9 | 5.38 | -8.29 |
| AlFe_4_ | mp-1228952 | $Fmmm$ | 0.800 |  | Y | 20 (12×9×2) | 120 (2×3×1) | 1.76 | 12.047 | 157.4 | 4.61 | -6.09 |
| BCC Fe | mp-13 | $Im\bar{3}m$ | 1.000 | Y | Y | 2 (31×31×31) | 54 (5×5×5) | 2.23 | 11.438  11.84^a^ | 188.5  173.1^a^ | 5.89 | 0.00 |

^a^ Experimental data of pure elements collected by Shang et al. ^7^; where the bulk moduli were calculated from elastic constants *C*_ij_ at 0 K or 4.2 K.

^b^ Measured data ^8^.

^c^ Determined by single crystal X-ray diffraction ^5^.

^d^ All possible Al_5_Fe_2_ configurations were generated by the ATAT code ^9^ using the 24-atom supercell with Al partially in Wyckoff sites 4b (occupation of 0.32) and 8f (occupation of 0.24) of space group $Cmcm$ ^10^. Only one or two of the low energy configurations were used herein after DFT calculations for each composition.

^e^ Starting from the configuration of Al_5_Fe_2_ (5), the USPEX method ^11^ was used to find the lowest energy configuration; see the relaxed structure in VASP POSCAR formation in **Table S 2**.

^f^ Measured data of Al_5_Fe_2_ ^10^.

^g^ Suggested low energy configurations of Al_5_Fe_2_ by Vinokur et al. ^6^.

^h^ Measured data of tetragonal Al_2_Fe which was synthesized at 7.5 GPa and 873 K ^12^.

^i^ Based on one of the Wyckoff sites, 2i of $P\bar{1}$, is occupied by both Fe (occupation of 0.705) and Al (occupation of 0.705) ^13^, the ATAT code ^9^ in terms of the 38- and 57-atom supercells of Al_2_Fe was used to build all possible configurations, and only the low configurations were used herein.

^j^ Measured data of Al_2_Fe ^13^.

^k^ Measured data at 1120 °C for Al_8_Fe_5_ and room temperature for AlFe (B2) ^14^.

^m^ Based on the measured lattice parameter of 5.791Å ^15^.

Table S 2. Predicted low energy configuration of Al_5_Fe_2_ by the USPEX method [48]; showing in the POSCAR format used by VASP.

| Al5Fe2 obtained from USPEX  1.00000000000000  3.9856825100556255 0.0180139195117352 -0.0119959383759500  -2.0223150583039149 6.3609873531164363 -0.1236273035337276  0.0309977658533041 -1.8398680209679523 7.4966967430280356  Al Fe  10 4  Direct  0.3957394519960999 0.4091982017268107 0.2399459199414556  0.9778201474046904 0.5733094819459010 0.7894016211923789  0.6007990938587022 0.8193711169069814 0.7272403546970154  0.3741398967557212 0.3659567715349421 0.8905644993630734  0.7931962793206077 0.2041882284349636 0.4876203816651489  0.7853874596828249 0.1884574927561469 0.0291626674869105  0.9931096244929684 0.6040178111125385 0.4407466632656956  0.5855374362993363 0.7886233363379547 0.0758845690733351  0.2043975531648467 0.0266110548503117 0.6260544053994812  0.1829370190242693 0.9835634314595856 0.2767080212576478  0.4026563358045122 0.4230849778281406 0.5685866454611316  0.9815066050839097 0.5805820997532304 0.1100919446767003  0.5971753624837085 0.8120861753909097 0.4065897199004853  0.1759887346277932 0.9696118199616005 0.9480645866195526 |
| --- |

Table S 3. Predicted elastic properties (GPa or dimensionless) of the Al-Fe IMCs, including single crystal elastic constants *C*_ij_ and the calculated aggregate properties in Hill approach (H) ^16^ of bulk modulus (*B*_H_), shear modulus (*G*_H_), *B*_H_/*G*_H_ ratio, Poisson’s ratio (ν_H_), and anisotropy index *A*^U^ (*A*^U^ = 0 for locally isotropic single crystals); see details of the methodology in ^7^. It shows that Al_8_Fe_5_ is very close to an isotropic crystal, while AlFe_3_ (L1_2_) shows extremely anisotropic.

| Materials | *C*_ij_ matrix (only the upper values) | *B*_H_ | *G*_H_ | *B*_H_/*G*_H_ | ν_H_ | *A*^U^ |
| --- | --- | --- | --- | --- | --- | --- |
| Al  (FCC, Cubic)^a^ | $\left[ \begin{matrix} 98.3 & 67.6 & 67.6 & 0 & 0 & 0 \\ & 98.3 & 67.6 & 0 & 0 & 0 \\ & & 98.3 & 0 & 0 & 0 \\ & & & 25.7 & 0 & 0 \\ & & & & 25.7 & 0 \\ & & & & & 25.7 \end{matrix} \right]$ | 77.8  77.8^b^ | 20.9 | 3.728 | 0.377 | 0.329 |
| Al_6_Fe  (Orth.) | $\left[ \begin{matrix} 240.4 & 43.3 & 68.8 & 0 & 0 & 0 \\ & 185.2 & 82.8 & 0 & 0 & 0 \\ & & 149.3 & 0 & 0 & 0 \\ & & & 56.8 & 0 & 0 \\ & & & & 32.6 & 0 \\ & & & & & 65.7 \end{matrix} \right]$ | 106.6  105.2^b^ | 53.0 | 2.013 | 0.287 | 0.694 |
| Al_13_Fe_4_  (Mono.) | $\left[ \begin{matrix} 241.6 & 93.4 & 65.8 & 0 & -1.7 & 0 \\ & 216.9 & 51.3 & 0 & 10.2 & 0 \\ & & 254.3 & 0 & -7.6 & 0 \\ & & & 69.2 & 0 & 1.0 \\ & & & & 78.4 & 0 \\ & & & & & 80.5 \end{matrix} \right]$ | 125.6  121.4^b^ | 76.7 | 1.639 | 0.247 | 0.163 |
| Al_5_Fe_2_ (5)  (Based on  orth.) | $\left[ \begin{matrix} 220.6 & 82.5 & 100.0 & 0 & 0 & 0 \\ & 237.3 & 48.4 & 0 & 0 & 0 \\ & & 277.3 & 0 & 0 & 0 \\ & & & 83.6 & 0 & 0 \\ & & & & 102.2 & 0 \\ & & & & & 92.6 \end{matrix} \right]$ | 132.6  126.8^b^ | 87.8 | 1.509 | 0.229 | 0.177 |
| Al_5_Fe_2_ (6)  (by USPEX,  mono.) | $\left[ \begin{matrix} 275.9 & 107.0 & 64.7 & 17.5 & -6.5 & 0 \\ & 253.8 & 47.3 & 0 & -7.2 & 0 \\ & & 274.4 & -21.6 & -6.6 & 0 \\ & & & 53.5 & 0 & 0 \\ & & & & 79.2 & -7.0 \\ & & & & & 116.2 \end{matrix} \right]$ | 137.0  132.4^b^ | 83.7 | 1.638 | 0.247 | 0.664 |
| Al_2_Fe  (MoSi_2_-type,  tetr.) | $\left[ \begin{matrix} 274.5 & 89.9 & 110.6 & 0 & 0 & 0 \\ & 274.5 & 110.6 & 0 & 0 & 0 \\ & & 223.1 & 0 & 0 & 0 \\ & & & 168.9 & 0 & 0 \\ & & & & 168.9 & 0 \\ & & & & & 130.7 \end{matrix} \right]$ | 154.7  147.2^b^ | 115.8 | 1.336 | 0.201 | 0.812 |
| Al_2_Fe (1)  (Triclinic) | $\left[ \begin{matrix} 243.9 & 79.1 & 72.4 & 0.0 & -13.2 & 0.0 \\ & 258.7 & 85.5 & 4.6 & 8.9 & 2.0 \\ & & 256.8 & -8.4 & 10.1 & 1.6 \\ & & & 77.4 & 11.7 & -4.2 \\ & & & & 85.1 & -4.1 \\ & & & & & 85.3 \end{matrix} \right]$ | 136.9  125.6^b^ | 83.3 | 1.643 | 0.247 | 0.134 |
| Al_2_Fe (2)  (Triclinic) | $\left[ \begin{matrix} 241.6 & 75.6 & 72.5 & 0.0 & -14.3 & 0.6 \\ & 248.4 & 88.3 & 6.9 & 9.1 & 1.6 \\ & & 247.1 & -10.1 & 7.1 & 5.5 \\ & & & 75.3 & 11.5 & -4.9 \\ & & & & 83.2 & -3.6 \\ & & & & & 79.5 \end{matrix} \right]$ | 134.3  125.6^b^ | 79.8 | 1.682 | 0.252 | 0.147 |
| Al_8_Fe_5_  (D8_2,_ cubic) | $\left[ \begin{matrix} 241.1 & 92.1 & 92.1 & 0 & 0 & 0 \\ & 241.1 & 92.1 & 0 & 0 & 0 \\ & & 241.1 & 0 & 0 & 0 \\ & & & 72.6 & 0 & 0 \\ & & & & 72.6 & 0 \\ & & & & & 72.6 \end{matrix} \right]$ | 141.8  138.0^b^ | 73.3 | 1.933 | 0.279 | 0.001 |
| AlFe  (B2, cubic) | $\left[ \begin{matrix} 265.6 & 143.3 & 143.3 & 0 & 0 & 0 \\ & 265.6 & 143.3 & 0 & 0 & 0 \\ & & 265.6 & 0 & 0 & 0 \\ & & & 145.0 & 0 & 0 \\ & & & & 145.0 & 0 \\ & & & & & 145.0 \end{matrix} \right]$ | 184.0  174.3^b^ | 93.6 | 1.795 | 0.265 | 0.951 |
| AlFe_3_  (L1_2,_ cubic | $\left[ \begin{matrix} 204.5 & 180.1 & 180.1 & 0 & 0 & 0 \\ & 204.5 & 180.1 & 0 & 0 & 0 \\ & & 204.5 & 0 & 0 & 0 \\ & & & 135.5 & 0 & 0 \\ & & & & 135.5 & 0 \\ & & & & & 135.5 \end{matrix} \right]$ | 188.3  169.1^b^ | 56.5 | 3.330 | 0.367 | 11.040 |
| AlFe_3_  (D0_3,_ cubic) | $\left[ \begin{matrix} 240.3 & 175.7 & 175.7 & 0 & 0 & 0 \\ & 240.3 & 175.7 & 0 & 0 & 0 \\ & & 240.3 & 0 & 0 & 0 \\ & & & 147.4 & 0 & 0 \\ & & & & 147.4 & 0 \\ & & & & & 147.4 \end{matrix} \right]$ | 197.2  164.1^b^ | 81.0 | 2.433 | 0.321 | 3.343 |
| Fe  (BCC, cubic)^a^ | $\left[ \begin{matrix} 260.7 & 140.5 & 140.5 & 0 & 0 & 0 \\ & 260.7 & 140.5 & 0 & 0 & 0 \\ & & 260.7 & 0 & 0 & 0 \\ & & & 89.8 & 0 & 0 \\ & & & & 89.8 & 0 \\ & & & & & 89.8 \end{matrix} \right]$ | 180.6  173.1^b^ | 76.5 | 2.362 | 0.315 | 0.196 |

^a^Measured elastic constants for Al at 0 K (*C*_11_ =114.3, *C*_12_ = 61.9 GPa, and *C*_44_ = 31.6 GPa) and Fe at 4.2 K (*C*_11_ =243.1, *C*_12_ = 138.1 GPa, and *C*_44_ = 121.9 GPa) ^16^.

^b^ Predicted value by EOS fitting, see **Table S 1**.

**References of Supplementary Material:**

1. Wolverton, C. & Ozoliņš, V. Entropically favored ordering: The metallurgy of Al_2_Cu revisited. *Phys. Rev. Lett.* **86**, 5518–5521 (2001).

2. Shang, S.-L., Wang, Y., Du, Y. & Liu, Z.-K. Entropy favored ordering: Phase stability of Ni_3_Pt revisited by first-principles. *Intermetallics* **18**, 961–964 (2010).

3. Wang, K. *et al.* Unveiling non-equilibrium metallurgical phases in dissimilar Al-Cu joints processed by vaporizing foil actuator welding. *Mater. Des.* **186**, 108306 (2020).

4. Jain, A. *et al.* Commentary: The Materials Project: A materials genome approach to accelerating materials innovation. *APL Mater.* **1**, 011002 (2013).

5. Popčević, P. *et al.* Anisotropic physical properties of the Al_13_Fe_4_ complex intermetallic and its ternary derivative Al_13_(Fe,Ni)_4_. *Phys. Rev. B* **81**, 184203 (2010).

6. Vinokur, A. I., Hilleke, K. P. & Fredrickson, D. C. Principles of weakly ordered domains in intermetallics: The cooperative effects of atomic packing and electronics in Fe_2_Al_5_. *Acta Crystallogr. Sect. A* **75**, 297–306 (2019).

7. Shang, S. L. *et al.* First-principles calculations of pure elements: Equations of state and elastic stiffness constants. *Comput. Mater. Sci.* **48**, 813–826 (2010).

8. Aliravci, C. A. & Pekgüleryüz, M. Ö. Calculation of phase diagrams for the metastable Al-Fe phases forming in direct-chill (DC)-cast aluminum alloy ingots. *Calphad* **22**, 147–155 (1998).

9. van de Walle, A. *et al.* Efficient stochastic generation of special quasirandom structures. *Calphad* **42**, 13–18 (2013).

10. Burkhardt, U., Grin, Y., Ellner, M. & Peters, K. Structure refinement of the iron–aluminium phase with the approximate composition Fe_2_Al_5_. *Acta Crystallogr. Sect. B* **50**, 313–316 (1994).

11. Lyakhov, A. O., Oganov, A. R., Stokes, H. T. & Zhu, Q. New developments in evolutionary structure prediction algorithm USPEX. *Comput. Phys. Commun.* **184**, 1172–1182 (2013).

12. Tobita, K. *et al.* Phase stability and thermoelectric properties of semiconductor-like tetragonal FeAl_2_. *Sci. Technol. Adv. Mater.* **20**, 937–948 (2019).

13. Chumak, I., Richter, K. W. & Ehrenberg, H. Redetermination of iron dialuminide, FeAl_2_. *Acta Crystallogr. Sect. C* **66**, i87–i88 (2010).

14. Stein, F., Vogel, S. C., Eumann, M. & Palm, M. Determination of the crystal structure of the ε phase in the Fe-Al system by high-temperature neutron diffraction. *Intermetallics* **18**, 150–156 (2010).

15. Nishino, Y. *et al.* Semiconductorlike behavior of electrical resistivity in Heusler-type Fe2VAl Compound. *Phys. Rev. Lett.* **79**, 1909–1912 (1997).

16. Simmons, G. & Wang, H. *Single crystal elastic constants and calculated aggregate properties: A handbook*. (M.I.T. Press, 1971).
